# Automatic Assessment of Loneliness in Older Adults Using Speech Analysis on Responses to Daily Life Questions

**DOI:** 10.3389/fpsyt.2021.712251

**Published:** 2021-12-13

**Authors:** Yasunori Yamada, Kaoru Shinkawa, Miyuki Nemoto, Tetsuaki Arai

**Affiliations:** ^1^IBM Research, Tokyo, Japan; ^2^Dementia Medical Center, University of Tsukuba Hospital, Tsukuba, Japan; ^3^Division of Clinical Medicine, Department of Psychiatry, Faculty of Medicine, University of Tsukuba, Tsukuba, Japan

**Keywords:** health-monitoring, speech analysis and processing, mental health, voice, social connectedness

## Abstract

Loneliness is a perceived state of social and emotional isolation that has been associated with a wide range of adverse health effects in older adults. Automatically assessing loneliness by passively monitoring daily behaviors could potentially contribute to early detection and intervention for mitigating loneliness. Speech data has been successfully used for inferring changes in emotional states and mental health conditions, but its association with loneliness in older adults remains unexplored. In this study, we developed a tablet-based application and collected speech responses of 57 older adults to daily life questions regarding, for example, one's feelings and future travel plans. From audio data of these speech responses, we automatically extracted speech features characterizing acoustic, prosodic, and linguistic aspects, and investigated their associations with self-rated scores of the UCLA Loneliness Scale. Consequently, we found that with increasing loneliness scores, speech responses tended to have less inflections, longer pauses, reduced second formant frequencies, reduced variances of the speech spectrum, more filler words, and fewer positive words. The cross-validation results showed that regression and binary-classification models using speech features could estimate loneliness scores with an *R*^2^ of 0.57 and detect individuals with high loneliness scores with 95.6% accuracy, respectively. Our study provides the first empirical results suggesting the possibility of using speech data that can be collected in everyday life for the automatic assessments of loneliness in older adults, which could help develop monitoring technologies for early detection and intervention for mitigating loneliness.

## 1. Introduction

Loneliness is a subjective and perceived state of social and emotional isolation. Importantly, loneliness is a specific construct that is associated with but distinguished from depression, anxiety, and objective social isolation. As the world's elderly population increases, loneliness in older adults is becoming a serious health problem. In older adults, loneliness has been prospectively associated with a wide range of adverse health outcomes including morbidity and mortality (1, 2), function decline (3), depression (4, 5), cognitive decline (6, 7), and incidents of dementia, especially Alzheimer's disease (8, 9). A meta-analysis has shown that loneliness increases the risk of mortality comparable with other well-known risk factors, such as smoking, obesity, and physical inactivity (10). Moreover, the increases in the aging population and prevalence of loneliness make loneliness a more serious social and health problem (11, 12). In fact, the prevalence of loneliness has increased from an estimated 11–17% in the 1970s (11, 13) to about 20–40% for older adults (1, 14, 15). From these perspectives, a growing body of research have actively investigated possible interventions to reduce the prevalence of loneliness and its harmful consequences (11, 12, 16), and early detection of loneliness is urgently needed. One of the simplest ways is to use a direct question such as “Do you feel lonely?” but it has been reported to lead to underreporting due to the stigma associated with loneliness (17–19). Instead, multidimensional scales without explicitly using the word “lonely” [e.g., the 20-item UCLA Loneness Scale (20)] have been widely used for measuring loneliness in older adults (17). If loneliness measured by such a multidimensional scale can be automatically estimated by using passively collected data without requiring individuals to perform any task, this would help early detection of lonely individuals through frequent assessments with less burden on older adults.

Several studies have reported the possibility of automatic assessment of loneliness by using daily behavioral data (21–23). For example, one study collected behavioral data using in-home sensors such as time out-of-home and number of calls from 16 older adults for 8 months, and reported that a regression model using them could estimate scores of the UCLA Loneliness Scale with a correlation of 0.48 (21). Another study collected behavioral data including mobility, social interactions, and sleep from the smartphones and Fitbits of 160 college students and reported that a binary-classification model using these behavioral data could detect individuals with high loneliness scores at 80.2% accuracy (22). Although they suggested that the loneliness may produce measurable changes in daily behaviors and be automatically assessed by using these behavioral data, the behavioral types investigated in previous studies as well as studies researching these behavioral types still remains limited. Being capable of assessing loneliness using various types of daily behaviors would help improve performance and extend the application scope.

Speech is an attractive candidate for automatically assessing loneliness. There is growing interest in using speech data for healthcare applications (24, 25), due to the improvement in audio quality recorded by portable devices and the popularity of voice-based interaction systems such as voice assistants in smart speakers and smartphones. For example, a number of studies used phone conversations passively recorded (26, 27) and others used speech responses to tasks with mobile devices (28–32). If automatic assessment of loneliness is possible using speech responses collected in either way (i.e., conversations with other people or speech responses collected through voice interfaces), it would greatly increase the opportunity and accessibility of assessment for early detection of loneliness.

Speech data has been used for capturing changes in various types of emotional states and mental health conditions including depression (33–41), suicidality (35, 42), and bipolar disorder (27, 43). As a result of a complexity of the speech production process involving motor, cognitive, and physiological factors, speech has been thought to be a sensitive output system such that changes in individuals' emotional states and mental health conditions can produce measurable acoustic, prosodic, and linguistic changes (35, 44, 45). Studies have shown the promise in using speech as an objective biomarker for detecting/predicting mental illness (46, 47) and monitoring a patient's symptoms (48, 49). For example, previous studies on depressive speech reported substantial changes in acoustic, prosodic, and linguistic features including reduced formant frequencies (35, 36, 40), reduced pitch variation (less inflections) (41, 50), more pauses (34, 38), more negative words, and fewer positive words (33, 37). Although there has been no study investigating the relationship between loneliness and speech data that can be collected in everyday situations, it is reasonable to explore the possibility that speech data could be used for assessing loneliness in older adults.

We aimed to investigate whether speech features associated with loneliness levels in older adults can be found in speech data that can be collected in everyday life and whether these speech data can be used for estimating loneliness levels and detecting individuals with higher levels of loneliness. To this end, we developed a tablet-based application and collected speech responses to daily life questions regarding, for example, one's feelings and future travel plans. We also collected self-rated scores of the UCLA Loneliness Scale from the same participants. From audio data of the speech responses, we automatically extracted speech features characterizing acoustic, prosodic, and linguistic aspects, and investigated the association between these speech features and loneliness scores using correlation analysis and machine learning models.

## 2. Methods

### 2.1. Participants

We recruited healthy older adults through local recruiting agencies or advertisements in the local community in Ibaraki, Japan. Participants were excluded if they had self-reported diagnoses of mental illness at the time of recruitment (e.g., major depression, bipolar disorder, and schizophrenia), had self-reported prior diagnoses of neurodegenerative diseases (e.g., Parkinson's disease, dementia, and mild cognitive impairment), or had other serious diseases or disabilities that would interfere with the assessments of this study. All examinations were conducted in Japanese. This study was conducted under the approval of the Ethics Committee, University of Tsukuba Hospital (H29-065). All participants provided written informed consent after the procedures of the study had been fully explained.

In addition to the speech data collection and loneliness survey, all participants underwent the Mini-Mental State Examination to assess global cognition and Geriatric Depression Scale to assess depressive symptoms conducted by neuropsychologists. They also answered self-report instruments about their education level and marital status.

A total of 57 older individuals completed the speech data collection and loneliness survey [30 women (52.6%); 62–81 years; mean (SD) age, 73.2 (4.5) years; Table 1]. Table 1 summarizes the information about participant characteristics.

**Table 1:** Characteristics of study participants (*N*=57).

**Characteristics**		
Age [years], mean (SD)	73.2	(4.5)
Sex, n (%)		
Men	27	(47.4)
Women	30	(52.6)
Education [years], mean (SD)	13.8	(2.2)
Marital status, n (%)		
Never married	0	(0)
Divorced	2	(3.5)
Widowed	8	(14.0)
Married	47	(82.5)
Mini-Mental State Examination^a^, mean (SD)	27.4	(1.9)
Geriatric Depression Scale^b^, mean (SD)	2.9	(2.5)
UCLA Loneliness Score^c^, mean (SD)	37	(8.6)

a *The total possible score ranges from 0 to 30*.

b *The total possible score ranges from 0 to 15*.

c *The total possible score ranges from 20 to 80*.

### 2.2. Loneliness Survey

Loneliness levels for our participants were measured by the Japanese version of the UCLA Loneliness Scale version 3 (20, 51). This scale is a validated, self-rated instrument designed to measure feelings of emotional loneliness in a wide group of respondents, including older adults, and implemented in numerous epidemiologic studies of aging (3, 52, 53). It consists of 20 Likert-type questions on a four-point scale from “never” to “always”. The total score ranges from 20 to 80 with a higher score indicating greater loneliness, and there is no identified cut-off score that defines loneliness (54). We used this total score for the analysis.

### 2.3. Speech Data Collection

**Figure 1:**
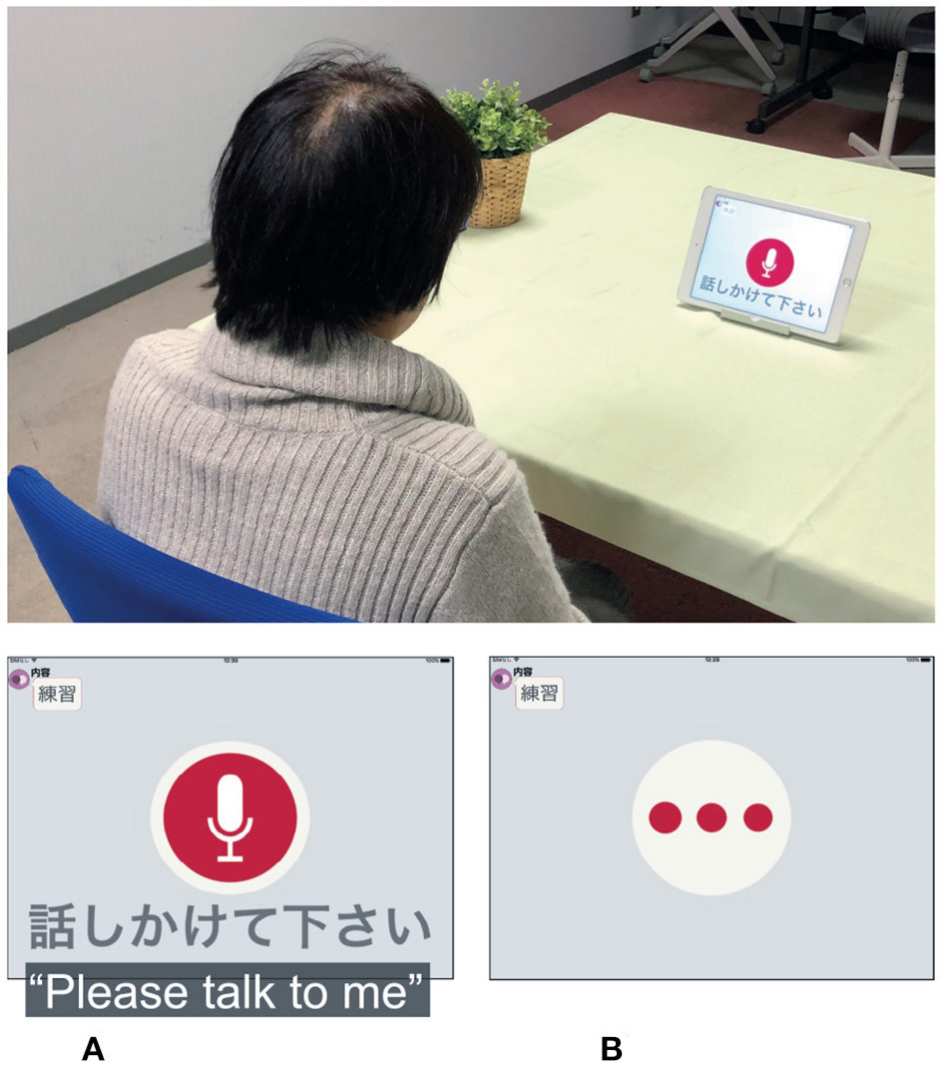
Overview of experimental setup for collecting speech data. **(A)** Participant's turn and **(B)** tablet's turn.

Participants sat down in front of the tablet and answered questions presented by a voice-based application on the tablet in a quiet room with low reverberation. The participants were asked to speak as naturally as possible. The tablet indicated whether it was speaking or listening (Figure 1). We used an iPad Air 2 and recorded voice responses by using the iPad's internal microphone (core audio format, 44,100 Hz, 16-bit).

The participants were asked eight daily life questions. The first two questions were frequently-used ones in daily conversations, that is, how one feels today and one's sleep quality last night. The next three questions were related to past experiences in terms of recalling old memories about a fun childhood activity as well as recent memories related to what was eaten for dinner yesterday and the day before yesterday. The next two questions were related to future expectations in terms of risk planning, such as one's response plans for an earthquake, and travel planning where participants chose one option from among two regarding future travel destinations and gave three reasons for their choice. The final question was related to general knowledge where participants explained a Japanese traditional event. For the actual sentences of the daily life questions, please see Supplementary Table 1.

### 2.4. Speech Data Analysis

From the speech responses of each participant to the eight questions, we automatically extracted a total of 160 speech features consisting of 128 acoustic features, 16 prosodic features, and 16 linguistic features. These features were determined on the basis of previous studies on inferring changes in emotional states and mental health conditions such as depression and suicidality (27, 33–43, 55–57). Full list of speech features is available in Supplementary Table 2.

As a preprocessing step, we first converted the audio data of each response into text data (i.e., automatic speech recognition) and divided the audio signals into voice and silence segments (i.e., voice activity detection) by using the IBM Watson Speech to Text service. All acoustic and prosodic features were extracted from the audio signals of voice segments, except for pause-related features, which were calculated by using the time duration of silence segments. Linguistic features were extracted from the text data after word tokenization, part-of-speech tagging, and word lemmatization using the Japanese morphological analyzer Janome (version 0.3.10 ^1^) in Python (version 3.8).

The acoustic features consisted of two feature types related to formant frequencies and Mel-frequency cepstral coefficients (MFCCs). Formant frequencies contain information related to acoustic resonances of the vocal tract and thus are thought to be able to capture changes in vocal tract properties affected by both an increase in muscle tension and changes in salivation and mucus secretion due to mental state changes (35). For example, the first and second formant (F1 and F2) are mainly associated with the tongue position: the F1 frequency is inversely related to the height of the tongue, while the F2 frequency is directly related to the frontness of the tongue position (58, 59). Limited movements of the articulators and particularly of the tongue, for example due to increased muscle tension, lead to inadequate vowel formation characterized by a lowering of normally high frequency formants and by an elevation of normally low frequency formants (60). Decreased formant frequencies were reported with increasing levels of speaker depression (35, 36, 40), and formant-based features have been frequently used for detecting depressive speech (46, 47). In addition, MFCCs are spectral features characterizing the frequency distribution of a speech signal at specific time instance information and designed to take into account the response properties of the human auditory system (61). As with the formant-based features, MFCCs have consistently been observed to change with individuals' mental states (35), and have been successfully used for various speech tasks including emotion recognition (62, 63), mood detection (64), and detection of depression (49, 65). In particular, the variances of the derivative of MFCCs were reported to show a consist trend of negative correlations with depression severity (49, 50). These decreased temporal variations in MFCCs with increasing depressive severity are thought to capture monotony and dullness of speech in clinical descriptions (35, 49). We thus used the first two formant frequencies (F1 and F2) and the variances of the first order derivatives (Δ) of the first 14 MFCCs. Because these features were extracted from each response to the eight questions, we obtained (2 + 14) × 8 = 128 acoustic features for each participant. To extract them, we used the Python-based (version 3.8) audio processing library librosa [version 0.8.0 (66)].

Prosodic features such as rhythm, stress, and intonation in speech conveys important information regarding individual's mental states. Commonly-used examples include pitch variation (i.e., inflection) and pause duration. Multiple studies reported a reduced pitch variation and an increased pause duration in accordance with increasing levels of depression severity (34, 38) as well as brief emotion induction of sadness in normal participants (39), although a number of studies showed no substantial change (34, 67). We thus used pitch variation and pause duration for prosodic features. Specifically, we calculated the pitch variation and pause duration in all eight responses of each participants and used total 2 × 8 = 16 features as the prosodic features. For estimating pitch, we used fundamental frequency calculated with the Python-based audio processing library Signal_Analysis (version 0.1.26 ^2^).

The linguistic features consisted of three feature types related to positive words, negative words and filler words (e.g., “umm,” “hmm,” “uh”). Sentiment analysis has been one of most representative approaches to detect changes in mental health conditions from linguistic cues. For example, several studies reported that depressed individuals tended to use more negative words and fewer positive words than non-depressed individuals (33, 37). Filler words are commonly found in spontaneous speech and have been suggested as important signatures for detecting depression (55–57). We thus used the number of positive and negative words and the proportion of filler words as linguistic features. Specifically, we counted the number of positive and negative words, respectively, in each speech response to the four questions expected to include positive or negative words: questions about a fun childhood activity, response plan for an earthquake, future travel plans, and a Japanese traditional event. Each word was determined to be positive (or negative) by using the Japanese Sentiment Polarity Dictionary (68, 69). The number of filler words was obtained by counting two kinds of words: those estimated as hesitation by automatic speech recognition using the IBM Watson Speech to Text service and those defined as fillers in the Japanese IPA dictionary^3^. We thus used 2 × 4 + 1 × 8 = 16 linguistic features for each participant.

### 2.5. Statistical Analysis

Spearman's nonparametric rank correlation coefficient was computed to test the null hypothesis that there is no correlation between each speech feature and scores of the UCLA Loneliness Scale. We did not adjust for multiple comparisons, and P values below 0.05 were considered to be statistically correlated. The statistical analyses were conducted using the Statistics and Machine Learning Toolbox (version 11.1) for the MATLAB (version R2017a, The MathWorks Inc) environment.

### 2.6. Regression and Classification Models

**Figure 2:**
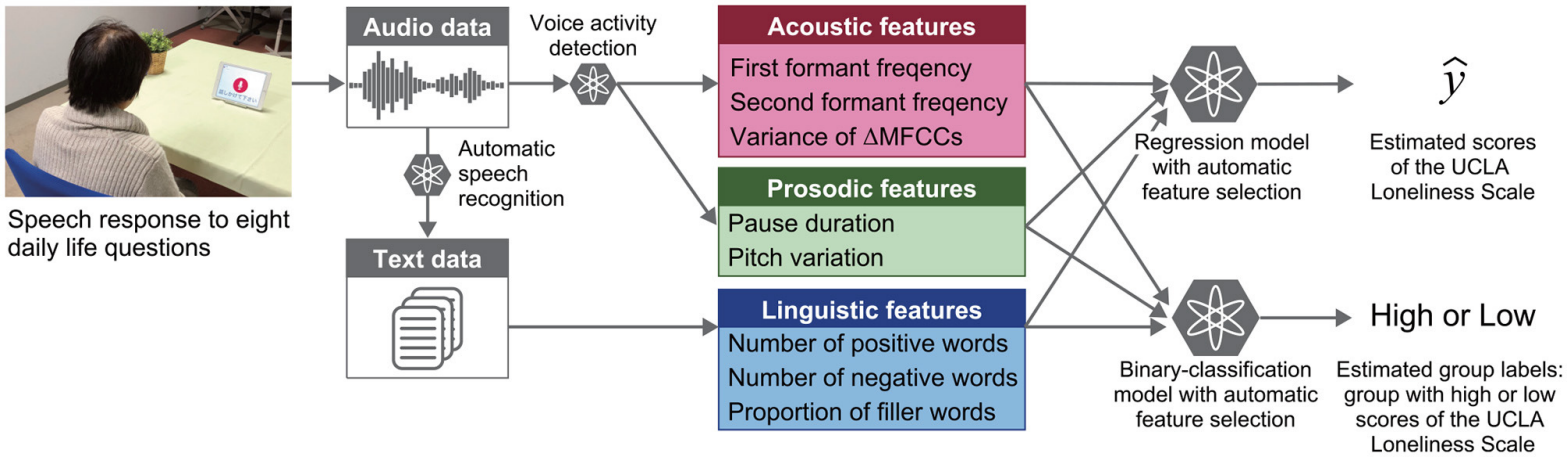
Overview of automatic analysis pipeline for estimating loneliness scores and for detecting individuals with high loneliness scores from speech responses to daily life questions.

The regression and binary-classification models were built to investigate whether speech features can be used for estimating scores of the UCLA Loneliness Scale and for detecting individuals with high scores, respectively (Figure 2). For the cut-off score for building the binary-classification model, because there is no designated cut-off score, we used the mean + 1SD of our participants' scores in the same manner as a previous study on characteristics of lonely older adults using the same UCLA Loneliness Scale (54). To facilitate interpretations and compare model performance with those of previous studies on automatic assessment of loneliness by using daily behavioral data (21–23), we focused on developing models using only speech features without other demographic information such as gender.

The regression and binary-classification models were built by using multiple types of machine learning models by combining them with automatic feature selection using a sequential forward selection algorithm. Model performances were evaluated by 20 iterations of 10-fold cross-validation methods. In the ten-fold cross-validation, the model was trained using 90% of the data (the “training set”) while the remaining 10% was used for testing. The process was repeated ten times to cover the entire span of the data, and the average model performance was calculated. Regression model performances were evaluated by using R^2^, explained variance (EV), mean absolute error (MAE), and root mean square error (RMSE). MAE and RMSE were calculated by the following equations: $\text{MAE}=1/n\sum_{i=1}^{n}\left|y_{i}-{\hat{y}}_{i}\right|$ and $\text{RMSE}=\sqrt{1/n\sum_{i=1}^{n}{\left(y_{i}-{\hat{y}}_{i}\right)}^{2}}$, where y_i_ and ${\hat{y}}_{i}$ are actual and estimated scores of the UCLA Loneliness Scale for the i-th participant, respectively. Binary-classification model performances were evaluated by accuracy, sensitivity, specificity, and F1 score. The total number of input features to the regression and binary-classification models was set to 48 so that the number of acoustic, prosodic, and linguistic features would be the same (i.e., 16× 3 = 48). The inputs of acoustic features were selected on the basis of absolute values of Spearman correlation coefficients with scores of the UCLA Loneliness Scale in the training set.

The machine learning models included k-nearest neighbors (70), random forest (RF) (71), and support vector machine (SVM) (72). The parameters that we studied were as follows: the number of neighbors for the k-nearest neighbors; the number and the maximum depth of trees for RF; kernel functions, penalty parameter, the parameter associated with the width of the radial basis function (RBF) kernel, class weights for the classification model, and the parameter of the regression model related to the loss function for the SVM. We used algorithms implemented using the Python package scikit-learn (version 0.23.2) and all other parameters were kept at their default values. We performed a grid search and determined the aforementioned parameters.

## 3. Results

**Figure 3:**
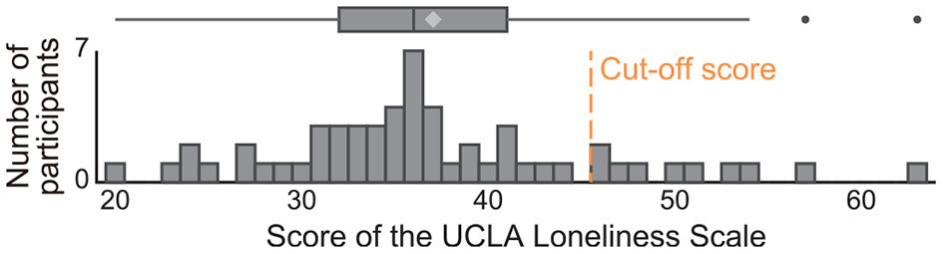
Histogram of scores of the UCLA Loneliness Scale for study participants. Cut-off score was determined by using the mean + 1SD of our participants' scores and 46 points. In our sample, 10 older adults (18% of the participants) scored equal to or greater than the cut-off score.

The mean score for the UCLA Loneliness Scale was 37.0 (SD: 8.6; range for participants, 20–63; possible range, 20–80; Figure 3). The Cronbach's alpha coefficient was 0.89. The cut-off score for dividing participants into two groups with low and high loneliness scores for building a binary-classification model was 46 points, which was determined by the mean + 1SD of our participants' scores in the same manner as that of a previous study (54). In our sample, ten older adults (6 males, 4 females; 18% of the participants) scored equal to or greater than the cut-off score. They were similar values reported in the previous study investigating 173 older adults: cut-off score was 48 points, and 19% of their participants scored equal to or greater than their cut-off score (54). In regard to the speech data, we obtained an average of 319.7 sec (SD: 108.5) of speech responses to the eight daily life questions. The average duration of responses to each question varied between 4.2 and 75.4 sec.

**Figure 4:**
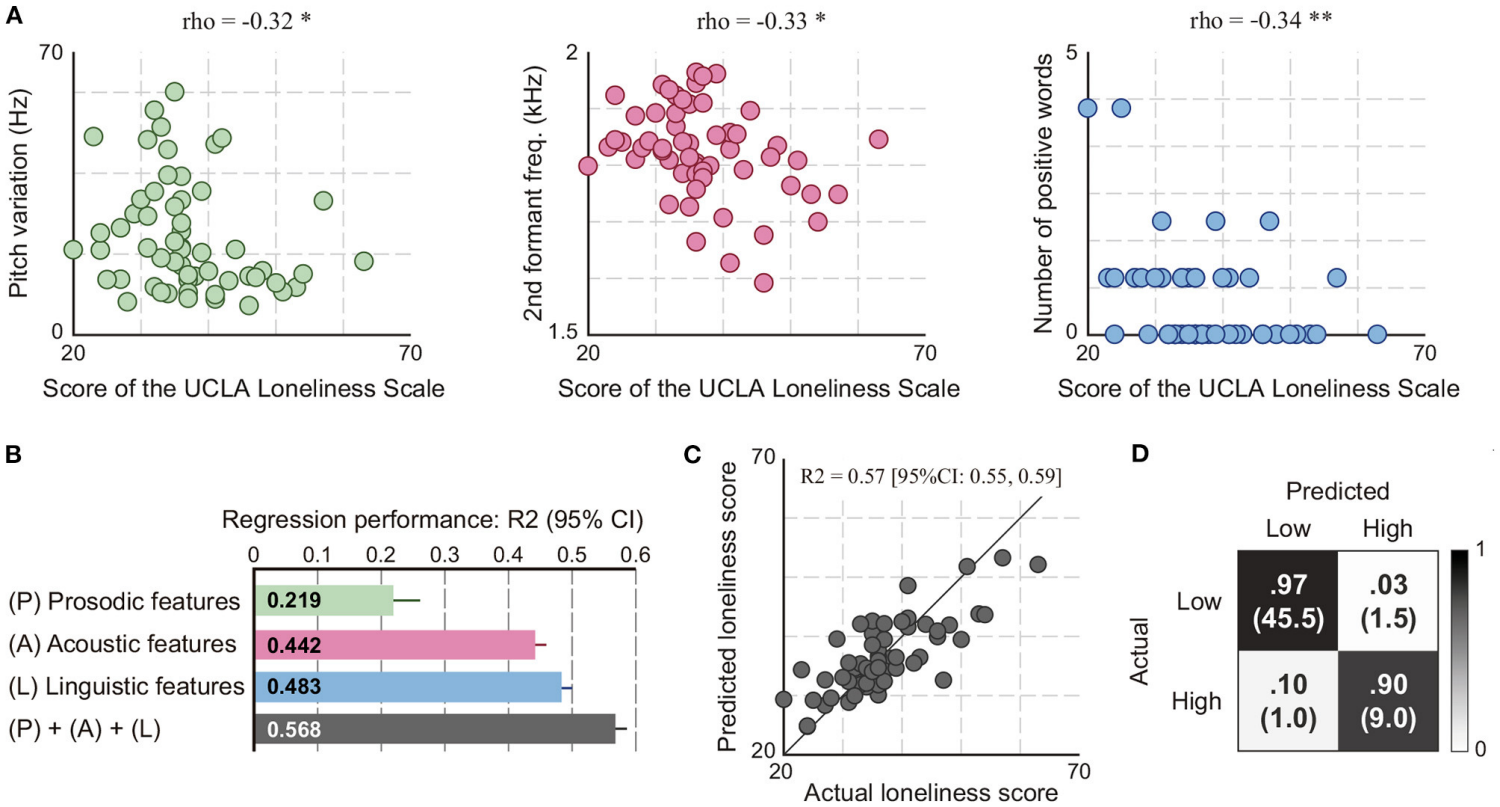
Analysis results of the associations of speech responses to eight daily life questions with scores of the UCLA Loneliness Scale. **(A)** Examples of speech features correlated with loneliness scores (Spearman correlation; *P < 0.05 and **P < 0.01). **(B)** Regression performances of the models using speech features for estimating loneliness scores. **(C)** Actual and predicted loneliness scores by the regression model using acoustic, prosodic, and linguistic features. **(D)** Confusion matrix of the binary-classification model using acoustic, prosodic, and linguistic features for detecting individuals with high loneliness scores. It was obtained using 20 iterations of 10-fold cross-validation. The number in parentheses indicates the mean number of participants among 20 iterations.

We first investigated associations of loneliness scores with each speech feature. Consequently, we found 21 speech features weakly correlated with loneliness scores (Spearman correlation ρ; 0.26 < |ρ| <0.41; P < 0.05; Supplementary Table 3): 15 acoustic features (13 features related to variance of ΔMFCCs and 2 features related to F2), 3 prosodic features (pitch variation and two features related to pause duration), 3 linguistic features (positive word frequency and two features related to filler words). With increasing loneliness scores, the acoustic features showed decreased F2 and reduced the variance of ΔMFCCs, the prosodic features showed decreased pitch variation and increased pause duration, and the linguistic features showed a decrease in the number of positive words and an increase in the proportion of filler words (Figure 4A and Supplementary Table 3). After controlling for age and sex as potential confounding factors (35, 73, 74), 15 of the 21 speech features remain correlated with loneliness scores (Supplementary Table 3).

We next built regression models using speech features to investigate whether speech response to daily life questions could be used for estimating scores of the UCLA Loneliness Scale. The result of iterative ten-fold cross validations showed that the model using speech features consisting of acoustic, prosodic, and linguistic features could estimate loneliness scores with an R^2^ of 0.568 (EV of 0.570, MAE of 4.46, and RMSE of 5.63) (Figures 4B,C and Table 2). This model was based on an SVM with an RBF kernel using 4 acoustic feature, 1 prosodic feature, and 3 linguistic features selected by the automatic feature selection procedure. The performances of this model calculated separately by sex were R^2^ of 0.599 (95% CI: 0.579 to 0.620) for women and R^2^ of 0.511 (95% CI: 0.487–0.535) for men. When building regression models separately by sex, the performances of the model for women and men were R^2^ of 0.648 (95% CI: 0.628–0.668) and R^2^ of 0.764 (95% CI: 0.744–0.784), respectively. We also built regression models separately using each acoustic, prosodic, and linguistic feature sets and compared their performances. Consequently, the model using linguistic features had the highest performance with an R^2^ of 0.483 (EV of 0.484, MAE of 4.75, and RMSE of 6.16) followed by that using acoustic features with an R^2^ of 0.442 (EV of 0.442, MAE of 4.86, and RMSE of 6.40), and that using prosodic features with an R^2^ of 0.219 (EV of 0.227, MAE of 5.96, and RMSE of 7.56) (Figure 4B and Table 2).

**Table 2:** Regression model performance of speech features predicting loneliness scores resulting from 20 iterations of 10-fold cross validation.

**Input variables**	**R^2^**	**EV**	**MAE**	**RMSE**
(P) Prosodic	0.219 [0.177, 0.261]	0.227 [0.185, 0.268]	5.96 [5.79, 6.12]	7.56 [7.36, 7.76]
(A) Acoustic	0.442 [0.424, 0.459]	0.442 [0.425, 0.460]	4.86 [4.78, 4.93]	6.40 [6.30, 6.50]
(L) Linguistic	0.483 [0.467, 0.500]	0.484 [0.468, 0.501]	4.75 [4.66, 4.83]	6.16 [6.06, 6.26]
(P) + (A) + (L)	0.568 [0.550, 0.586]	0.570 [0.553, 0.587]	4.46 [4.36, 4.57]	5.63 [5.51, 5.74]

*Each value indicates the average value across 20 iterations with 95% confidence interval. EV, explain variance; MAE, mean absolute error; RMSE, root mean square error*.

We finally investigated whether speech data could be used for detecting individuals with high loneliness scores by building a binary-classification model with speech features. The results of iterative ten-fold cross validations showed that the model using acoustic, prosodic, and linguistic features could detect individuals with high loneliness scores at 95.6% accuracy (90.0% sensitivity, 96.8% specificity, and 87.9% F1 score) (Figure 4D and Table 3). This model was based on an SVM with an RBF kernel using 5 acoustic features, 2 prosodic features, and 3 linguistic features. The performances of this model calculated separately by sex were 98.3% accuracy (95% CI: 97.1–99.5) for women and 92.6% accuracy (95% CI: 92.6–92.6) for men. When building binary-classification models separately by sex, the performances of the model for women and men were 100.0% accuracy (95% CI: 100.0–100.0) and 98.9% accuracy (95% CI: 98.1–99.7), respectively. For the models using the acoustic, prosodic, and linguistic feature sets separately, the results showed similar trends with those of the regression models: the model using acoustic features had the highest accuracy at 92.7% (95% CI: 92.2–93.3), followed by that using linguistic features with 91.0% accuracy (95% CI: 90.7–91.3), and that using prosodic features with 87.7% accuracy (95% CI: 87.7–87.7) (Table 3).

**Table 3:** Classification model performance of speech features detecting individuals with high loneliness level resulting from 20 iterations of 10-fold cross validation.

**Input variables**	**Accuracy (%)**	**Sensitivity (%)**	**Specificity (%)**	**F1 score (%)**
(P) Prosodic	87.7 [87.7, 87.7]	30.0 [30.0, 30.0]	100.0 [100.0, 100.0]	46.2 [46.2, 46.2]
(L) Linguistic	91.0 [90.7, 91.3]	60.0 [60.0, 60.0]	97.6 [97.2, 97.9]	70.0 [69.3, 70.7]
(A) Acoustic	92.7 [92.2, 93.3]	70.0 [70.0, 70.0]	97.6 [96.9, 98.2]	77.2 [75.9, 78.5]
(P) + (L) + (A)	95.6 [95.0, 96.2]	90.0 [90.0, 90.0]	96.8 [96.1, 97.6]	87.9 [86.4, 89.4]

*Each value indicates the average value across 20 iterations with 95% confidence interval*.

## 4. Discussion

We collected speech responses to eight daily life questions with our tablet-based application and investigated the associations of speech features automatically extracted from audio data of these speech responses with scores the UCLA Loneliness Scale. Our first main finding was that acoustic, prosodic, and linguistic characteristics each may have features affected by loneliness levels in older adults. Through correlation analysis, we could find acoustic, prosodic, and linguistic features correlated with loneliness scores. Our second finding was that the combination of acoustic, prosodic, and linguistic features could achieve high performances both for estimating loneliness scores and for detecting individuals with high loneliness scores. These findings showed the possibility of the use of speech responses usually observed in daily conversations (e.g., responses regarding today's feeling and future travel plans) for automatically assessing loneliness in older adults, which can help to promote future efforts toward developing applications for assessing and monitoring loneliness in older adults.

We found speech features correlated with loneliness scores in acoustic, prosodic, and linguistic characteristics in speech response to daily life questions. With increasing loneliness scores, speech responses tended to have less inflections and longer pauses in prosodic features; reduced second formant frequencies and variances of the speech spectrum (ΔMFCCs) in acoustic features; and fewer positive words and more filler words in linguistic features. All these trends in their changes were consistent with those observed in individuals with changes in emotional states and mental health conditions, especially those reported in previous studies on depressed speech [for F2 (35, 36, 40); for the variance of ΔMFCCs (49, 50); for pitch variation (41, 50); for pauses (34, 38); for positive words (33, 37); for filler words (55–57)]. This result may be reasonable because loneliness and depression are different constructs but closely correlated with each other (11). Considering similarities between loneliness and depression, including in their effects on speech characteristics, further studies including longitudinal data collection are required to ensure that the speech changes are due to either loneliness or depression and to identify changes in speech features particularly sensitive to loneliness rather than depression or mood. The potential mechanisms underlying the effects of loneliness on speech characteristics are poorly understood (75–77), but we may be able to explain them from the perspective of the associations of chronic psychological stress. Lonely individuals reported experiencing a great number of chronic stressors (78) and were more likely to perceive daily events as stressful (79, 80). Further, empirical studies suggested the associations of loneliness with exaggerated stress responses (75). These changes may potentially affect processes involved in the phonation and articulation muscular systems and speech production via changes to the somatic and autonomic nervous systems, which may result in producing measurable acoustic, prosodic, and linguistic changes (35). In this study, we observed the effects of loneliness on speech responses to daily life questions that were not designed to induce emotional responses, although we did not test effects of these questions on mood. This result suggest that loneliness may affect even daily speech through chronic psychological stress, although further research is needed. In addition, due to a complexity of loneliness, there are multiple scales for measuring loneliness from different viewpoints. For example, the UCLA Loneliness Scale is used in an attempt to measure loneliness as a global, unidimensional construct, while the de Jong Gierveld Loneliness Scale (81) is used to attempt to measure it as multifaceted phenomenon with separate emotional and social components (17). Therefore, investigating speech changes related to different loneliness scales may provide useful insights to deepening our understanding of the wide and complex profiles of loneliness.

The cross-validation results showed that the regression and binary-classification models using speech features could estimate loneliness scores with an *R*^2^ of 0.57 (Pearson correlation of 0.76) and detect individuals with high loneliness scores with 95.6% accuracy, respectively. Previous studies on assessments of loneliness using behavioral data focused on behavioral patterns such as phone usage, time out-of-home, step counts, and sleep duration, and they reported a regression performance with a correlation of 0.48 (21) and classification accuracy ranging from 80.2 to 91.7% accuracy (22, 23). Compared with their performance, both regression and classification models in our study showed better performances. Although there are differences in the methodology such as target population, cut-off scores, and number of samples, this improvement of model performance might come from the use of speech data instead of the behavioral patterns investigated in previous studies. Aligning with previous studies on the associations of speech with depression and suicidality, our results suggest that speech may be one of the key behavioral markers for automatically detecting and predicting changes in mental health conditions including loneliness in older adults. One of our contributions lies in providing the first empirical evidence showing the feasibility of using the automatic analysis of speech for detecting changes due to loneliness in older adults. In addition, many recent studies have explored the use of speech data for healthcare applications for monitoring various types of health statuses in older adults, for example, for detecting cognitive impairments (31, 82–84) and Alzheimer's disease (26, 28, 29, 32, 85–90), for detecting depression (38, 91, 92), and for predicting driving risks (30). Together with these previous studies, our results may help future efforts toward developing applications using speech data for automatically and simultaneously monitoring various types of health statuses including loneliness. On the other hand, these applications have raised numerous ethical concerns including informed consent, especially when using passive data, i.e., data generated without the active participation of the individual (e.g., GPS, accelerometer data, phone call) (93). Thus, the ethical implications need to be considered parallel to the development of these healthcare applications.

Comparing the model performances among speech feature types showed that acoustic features could achieve high accuracies comparable with linguistic features. In particular, for detecting individuals with high loneliness scores, the binary-classification model using acoustic features achieved the best accuracy. Although user-interface studies reported that voice input was effective and was preferable as an input modality for older adults (94–96), other studies reported that the performance of automatic speech recognition tended to be worse in older adults than in other age groups (97, 98). Because we analyzed only speech data collected in a lab setting, we may need to consider the possibility that there would be a situation where automatic speech recognition would be difficult to use for extracting linguistic features from speech data collected in living situations. In that case, our results may suggest that an approach focusing on developing a model for detecting individuals with high loneliness scores using paralinguistic features, especially acoustic features, would be useful and effective.

There were several limitations in this study. First, the number of questions was small and limited. Although our study provided the first empirical evidence of the usefulness of daily life questions for assessing loneliness in older adults, it still remains uninvestigated what kinds of daily conversations could particularly elicit changes associated with loneliness. To investigate this, data collection at home would be a good way to collect many speech responses by having participants using applications on a daily basis. Second, in terms of statistical analysis of correlation coefficients, we did not adjust for multiple comparisons across speech features due to the exploratory nature of this investigation. In addition, the results of a *post hoc* power analysis revealed that speech features except for the variance of ΔMFCC14 did not reach a power of 0.8 with a significance level of 0.05 (two-sided). A future study on larger samples should confirm our result about the effects of loneliness on speech characteristics. Third, residual confounding such as medication can still exist in addition to age and sex considered in the analysis (35). We also excluded individuals with diagnoses of mental illness such as major depression, because they may affect speech. Therefore, a further study using large samples with these confounding factors is required to further confirm our results about the usefulness of speech analysis for assessing loneliness. Fourth, the number of participants with higher loneliness scores was small and limited. This might affect the generalizability of our results. Finally, the results were obtained by analyzing speech data in Japanese. Thus, we need to investigate speech data in other languages to confirm our results regarding the usefulness of speech responses to daily life questions for assessing loneliness.

In summary, we provide the first empirical results suggesting the possibility of using the automatic analysis of speech responses to daily life questions for estimating loneliness scores and detecting individuals with high loneliness scores. The results presented in this work indicate that it could be feasible to automatically assess loneliness in older adults from daily conversational data, which can help promote future efforts toward the early detection and intervention for mitigating loneliness.

## Data Availability Statement

The datasets presented in this article are not readily available but derived and supporting data may be available from the corresponding author on reasonable request and with permission from the Ethics Committee, University of Tsukuba Hospital. Requests to access the datasets should be directed to Yasunori Yamada, ysnr@jp.ibm.com; Kaoru Shinkawa, kaoruma@jp.ibm.com.

## Ethics Statement

The studies involving human participants were reviewed and approved by the Ethics Committee, University of Tsukuba Hospital. The patients/participants provided their written informed consent to participate in this study.

## Author Contributions

YY and KS contributed to conception and design of the study and performed analysis and wrote the manuscript. YY, KS, and MN conducted the experiments. All authors have approved the final version.

## Funding

This work was supported by JSPS KAKENHI grant no. 19H01084.

## Conflict of Interest

YY and KS are employed by the IBM Corporation. The remaining authors declare that the research was conducted in the absence of any commercial or financial relationships that could be construed as a potential conflict of interest.

## Publisher's Note

All claims expressed in this article are solely those of the authors and do not necessarily represent those of their affiliated organizations, or those of the publisher, the editors and the reviewers. Any product that may be evaluated in this article, or claim that may be made by its manufacturer, is not guaranteed or endorsed by the publisher.

## Footnotes

^1^ https://mocobeta.github.io/janome/en/

^2^ https://brookemosby.github.io/Signal_Analysis/

^3^ https://ja.osdn.net/projects/ipadic/
