# Supplementary material for: Automatic Assessment of Loneliness in Older Adults Using Speech Analysis on Responses to Daily Life Questions
Source: Front Psychiatry. 2021 Dec 13;12:712251. doi: 10.3389/fpsyt.2021.712251 (PMC8710612; doi:10.3389/fpsyt.2021.712251)
Supplement: Supplementary file 1 [file Data_Sheet_1.PDF]

Supplementary Table 1. Actual sentences of daily life questions (translated from Japanese).

Daily life question	Actual sentences
Today’s feeling	“How are you doing today?”
Sleep quality last night	“Did you sleep well last night?”
A fun childhood activity	“Please explain one of the fun activities you engaged in when you were a child.”
Dinner menu for yesterday	“What did you eat for dinner yesterday?”
Dinner menu for the day before yesterday	“What did you eat for dinner the day before yesterday?”
Response plan for an earthquake	“If an earthquake strikes while you are at home, how would you act and in what order?”
Future travel destination	“If you could go on a trip this summer, where would you like to go, the mountains or the sea? Please choose one and give three reasons why you chose it.”
Japanese traditional event	“If you could explain the Shichi-Go-San to someone who doesn’t know about the event, how would you explain it?”

Supplementary Table 2. List of all speech features.

	Speech feature	Number of features
**Acoustic**	First formant frequency	8 (1 × 8)
	Second formant frequency	8 (1 × 8)
	Variance of ΔMFCC1-14	112 (14 × 8)
**Prosodic**	Pause duration	8 (1 × 8)
	Pitch variation	8 (1 × 8)
**Linguistic**	Number of positive words	4 (1 × 4)
	Number of negative words	4 (1 × 4)
	Proportion of filler words	8 (1 × 8)
**Total**		160

Note: Number of positive and negative words were extracted from each speech response to the four questions about a fun childhood activity, response plan for an earthquake, future travel plans, and a Japanese traditional event. Other features were extracted from each response to all eight questions.

Supplementary Table 3. Speech features correlated with loneliness scores. ρ: Spearman correlation. Δ: first order derivative.

Speech feature	Daily life question	*Unadjusted*		*Adjusted**	
		ρ	*P*-value	ρ	*P*-value
**Acoustic**					
Variance of ΔMFCC6	Dinner menu for yesterday	**-0.322**	**0.0147**	-0.239	0.0738
Variance of ΔMFCC6	Dinner menu for the day before yesterday	**-0.291**	**0.0279**	-0.233	0.0810
Variance of ΔMFCC7	A fun childhood activity	**-0.262**	**0.0493**	**-0.292**	**0.0273**
Variance of ΔMFCC8	Dinner menu for the day before yesterday	**-0.264**	**0.0476**	**-0.277**	**0.0372**
Variance of ΔMFCC10	A fun childhood activity	**-0.280**	**0.0349**	**-0.327**	**0.0132**
Variance of ΔMFCC13	Sleep quality last night	**-0.313**	**0.0179**	**-0.274**	**0.0391**
Variance of ΔMFCC13	Dinner menu for yesterday	**-0.294**	**0.0266**	**-0.286**	**0.0311**
Variance of ΔMFCC13	Future travel destination	**-0.289**	**0.0292**	**-0.319**	**0.0154**
Variance of ΔMFCC13	A fun childhood activity	**-0.287**	**0.0306**	**-0.290**	**0.0284**
Variance of ΔMFCC13	Response plan for an earthquake	**-0.273**	**0.0400**	**-0.274**	**0.0392**
Variance of ΔMFCC14	Sleep quality last night	**-0.405**	**0.0018**	**-0.438**	**0.0007**
Variance of ΔMFCC14	Response plan for an earthquake	**-0.272**	**0.0405**	**-0.296**	**0.0253**
Variance of ΔMFCC14	A fun childhood activity	**-0.262**	**0.0492**	**-0.304**	**0.0216**
Second formant frequency	Response plan for an earthquake	**-0.329**	**0.0124**	**-0.276**	**0.0380**
Second formant frequency	A fun childhood activity	**-0.316**	**0.0167**	-0.255	0.0554
**Prosodic**					
Pitch variation	Today's feeling	**-0.315**	**0.0170**	-0.215	0.1074
Pause duration	Dinner menu for the day before yesterday	**0.300**	**0.0233**	0.225	0.0931
Pause duration	Sleep quality last night	**0.288**	**0.0299**	0.203	0.1304
**Linguistic**					
Number of positive words	Response plan for an earthquake	**-0.345**	**0.0086**	**-0.307**	**0.0202**
Proportion of filler words	Response plan for an earthquake	**0.330**	**0.0122**	**0.308**	**0.0199**
Proportion of filler words	A fun childhood activity	**0.311**	**0.0184**	**0.321**	**0.0149**

* Adjusted for age and sex. Correlations at $P < 0.05$ in bold.
